# No Correlation Between Articulation Speed and Silent Reading Rate when Adults Read Short Texts

**DOI:** 10.5334/pb.1189

**Published:** 2023-07-17

**Authors:** Marc Brysbaert, Anke Vantieghem

**Affiliations:** 1 Department of Experimental Psychology, Ghent University, B-9000 Ghent, Belgium

**Keywords:** reading, phonological recoding, reading rate, speech rate

## Abstract

Silent reading often involves phonological encoding of the text in addition to orthographic processing. The nature of the phonological code is debated, however: Is it an abstract code or does it contain information about the pronunciation of the visual stimulus? To answer this question, we investigated the relationship between articulation speed and reading speed, both for silent reading and reading aloud. We investigated whether people with fast articulation speed read faster than people with slow articulation speed. We recruited 94 participants, who in a Zoom session were asked to read short texts silently or aloud. They were also asked to talk about their lives and say the numbers 1–10 or the months of the year as quickly as possible. Finally, they completed an online vocabulary test and an author recognition test. Multiple regression analysis and cluster analysis showed that although the speed of reading aloud and silent reading correlated to some extent, they belonged to two different clusters. Reading aloud was mainly related to talking fluency and articulation speed, while silent reading was more related to vocabulary and knowledge about fiction authors. These findings are consistent with the hypothesis that the phonological code in silent reading typically does not contain articulatory information, although our data do not rule out the possibility that this may be the case for a small percentage of people or when people read more difficult texts.

Silent text reading is rarely based on orthographic processing of the visual text alone. There are several findings indicating that readers also rely on phonological information (Brysbaert, 2022). Readers recode the visual information into an auditory code, which helps them understanding the text. Six findings point in this direction. First, many speakers have the impression of hearing an inner voice when they are reading silently (Perrone-Bertolotti et al., 2014). Second, people need more time to silently read sentences that are difficult to pronounce (tongue-twister effect; McCutchen & Perfetti, 1982). Third, letter detection is easier in stressed syllables than in unstressed syllables (e in introspect vs. e in apartment; Drewnowski & Healy, 1982). Fourth, readers often make homophone errors in semantic decision (misclassifying the written word “rows” as a flower; Van Orden, 1987). Fifth, people often overlook homophone errors in proofreading (missing the error in “they would meat for”; Daneman & Stainton, 1991). Finally, there is parafoveal homophone priming in silent reading. People read the word “rains” more quickly following parafoveal preview of “reins” than “ruins” (Pollatsek et al., 1992).

One reason why people rely on phonological information in addition to orthographic information is that silent reading is a very recent skill. Consequently, evolution has not yet had much chance to select genetic variations that are good at making connections between visual recognition of written scribbles and activating meaning information stored in long-term memory (Dehaene & Cohen, 2011; Van der Haegen et al., 2012). Until the 19th century, most people whispered while reading a text (Manguel, 1996). Indeed, most early research on silent reading investigated whether it was possible at all to read a text without recoding it aurally (Huey, 1908). Philosophers were convinced that human thinking depended on speech and that the visual code had to be transferred to auditory code before it could be understood. This process was called vocalisation, subvocalisation, or silent speech.

One line of research was to investigate whether readers activated their articulatory muscles when reading silently (nicely reviewed by Edfeldt, 1959). Curtis (1900), for instance, placed a tambourine on the larynx to record its movements. Of the 20 participants tested, 15 showed more movement in silent reading than in other types of mental activity. Only the curves for actual whispering gave more extreme results. Edfeldt (1959) repeated the study with a more sophisticated electromyograph, which allowed him to detect and record the electrical potential generated by articulatory muscle cells. He obtained data from 84 university students and reported greater signal during silent reading in 58 participants than in the control condition. In particular, students who scored low on four reading tests (involving reading speed, reading comprehension, intelligence, and vocabulary) showed increased activity. Activity was also higher for a difficult text than for an easy text.

Gradually, the question arose whether articulation is really necessary to aurally encode a visual text. This was based on research in which participants were asked to process visually presented words under articulatory suppression. If articulation is required to translate the visual code into a phonological code, it can be predicted that performance will decrease if participants have to continuously repeat a sequence like “thethethe” during the task. Besner (1987, p. 474) reviewed the literature and concluded that “when experimental manipulations force the use of a phonological code, it can be both derived from print and used as a basis for lexical access without any interference from [articulatory] suppression.” For instance, participants were as good deciding that the nonword pallis sounds like a real word (palace) under articulatory suppression as in a control condition without suppression. Therefore, Besner concluded that articulation was not needed to derive the phonological code from a visually presented word (see also Gupta & MacWhinney, 1995; Norris et al., 2018), a conclusion opposite to that of Edfeldt (1959). A limitation of Besner’s (1987) review was that it only addressed phonological recoding of words, not of a coherent text participants try to understand.

In a series of studies, Baddeley and colleagues investigated the effects of articulatory suppression on sentence reading (e.g. Baddeley & Lewis, 1981; Mattys et al., 2018). Their conclusion was that three memory codes are involved in silent reading: articulatory encoding, acoustic encoding, and visual encoding. Articulatory encoding is used to refresh information in the phonological loop and helps maintain word order, but is not required to access the meaning of individual words or short phrases and sentences. It is mainly used in cases of significant memory load (e.g. a difficult text, a need to memorise the text, …). The acoustic code is activated as part of visual word recognition and supports retrieval of information from the mental lexicon (see also Coltheart et al., 2001; Seidenberg, 2005). Finally, the visual code allows readers to directly activate semantic information based on the written words (see also Dijkstra et al., 2019; Ferrand & Grainger, 1994). Reading would be based mainly on orthographic and acoustic codes.

Another reason to think that articulation is not involved in phonological recoding, is that silent reading is much faster than speaking aloud. For a long time, it was believed that average reading rate in silent reading was 300 words per minute (wpm; Brysbaert, 2019). This is about twice as fast as the typical speech rate (140–180 wpm). The doubling of processing speed is easier to accept if one assumes that the phonological code used in silent reading is less detailed than the code needed for word pronunciation.

However, the speed argument for the absence of articulation in silent reading has recently been questioned. First, it was found that the average reading rate in silent reading is not 300 wpm but 240 wpm (Brysbaert, 2019). Second, it was found that auditory text understanding is rather good up to 270 wpm and that people may be able to articulate words at a speed of 240 wpm (Kuperman et al., 2021). So, in principle it is possible that people silently read at a speed equal to their fastest articulation rate and the rate at which they can understand spoken information (as also argued by Gagl et al. 2022).

A way to further address this question is not to look at average processing rates but at individual differences in reading and speaking rate. People differ reliably in the speed with which they read (see the results of our study below), in the speed with which they talk, and in how fast they can articulate a sequence of familiar words. Finding a correlation between these rates would constitute good evidence that a common code is involved. In contrast, not finding a significant correlation between the various measures would be more in line with the hypothesis that the phonological code in silent reading is more abstract than the articulatory code needed for speech. Surprisingly, no such study has yet been conducted.

In the present study, we measured several language processing speeds in the same participants. First, we included silent reading and reading aloud. If silent reading involves the same (articulatory) code as oral reading, we should find a statistically significant correlation between both speeds. Second, we also asked participants to talk for a few minutes, so that we could assess their usual talking rate. Significant correlations between speaking rate, silent and oral reading tell us that some processes are shared across the three tasks. The lack of significant correlations suggests that the tasks make use of different processes. Third, we collected information about the maximum articulation rate by asking participants to name the numbers 1–10 and the months of the year as fast as possible. In such a task, participants do not have to think about the message they are going to deliver and can focus entirely on their speech rate. If the inner speech in silent reading involves articulation, we expect a significant correlation between silent reading rate and articulation speed. Finally, we also collected information on participants’ vocabulary, their knowledge about fiction authors, and the number of books they had read in the previous year. These variables are known to correlate well with silent reading performance (Brysbaert et al., 2020; Vermeiren et al., 2023).

## Method

### Participants

Participants were adults between 18 and 60 years (see Table 1), as reading rate does not differ very much between these ages (Brysbaert, 2019). It was a snowball sample via word-of-mouth, starting with acquaintances of the first author. Participants were asked to complete a short online questionnaire and to indicate whether they were willing to take part in a study that would last about one hour.

**Table 1:** Descriptive statistics of the variables measured in this study.


	MIN.	1 QT	MED.	MEAN	3 QT	MAX.	SD

Age (yrs)	18	20	22	27.4	27	60	12.4

DART	0%	10%	14%	19%	23%	74%	14%

Books read	0	0	1	3.9	4	80	9.4

Vocabulary	13	22	27	27.5	33	40	7.3

Wpm silent reading	129	204	235	252.5	276	878	90.9

Wpm reading aloud	106	152	162	160.2	171	207	17.8

Wpm talking 1	127	160	176	175.9	191	237	22.6

Wpm talking 2	113	164	180	180.4	199	230	25.8

Wpm articulation digits	152	280	326	327.2	374	556	78.8

Wpm articulation months	104	147	164	164.4	182	235	26.0


DART = Dutch Author Recognition test, wpm = words per minute, QT = quartile, Med = median, SD = standard deviation.

The questionnaire asked for the participants’ age and gender, what their mother tongue was (to limit the study to native speakers of Dutch), and the level of education (primary education, secondary education, professional bachelor, academic bachelor, or academic master).

A total of 107 participants completed the questionnaire, of whom 94 agreed to take part in the study. Given COVID restrictions, testing happened online in 2020. The sample of 94 was the maximum we could reach and allowed us to measure correlations of r = .26 with 80% power. Of the 94 participants, 56 were women and all but five were still studying or had completed higher education (the remaining five stopped after high school). Due to the small sample size and the skewed distribution of education, no further use was made of these two person variables.

Participants were told in advance what tasks they would have to perform, that they would be filmed, that the videos would be seen only by the researcher with whom they interacted (AV), and that the videos would be deleted once numerical data on performance rate and accuracy were extracted. The participants signed an informed consent form, agreeing that their data could be used in scientific publications, provided the data are reported in anonymised form. The study followed the General Ethical Protocol of the Faculty of Psychology and Educational Sciences of Ghent University, in which the researchers promise to observe general ethical considerations (anonymity, informed consent, debriefing, the right to stop at any time, the right to be informed about the findings, no foreseen risk of harm, …).

### Tasks

#### Reading speed

To measure reading speed, we used 12 texts from Brysbaert et al. (2020). These were Dutch translations of texts originally compiled by Miller and Coleman (1967). They were short paragraphs of on average 150 words (256 syllables) describing an event or a topic. Six texts were read silently and six texts matched in average word length were read aloud. Difficulty level was assessed with T-Scan (Pander Maat et al., 2014) and ranged from B1 to C1 on a 6-category scale from A1 (easiest level) to C2 (most difficult level). These are the difficulty levels seen in newspaper articles. Average word length was 5.3 letters per word. The texts are available in Appendix 1. Dependent variable was the number of words read per minute (wpm).

#### Speaking rate

Speaking rate is the speed at which people usually talk, including stop words (like er), errors and error corrections. Participants were asked to talk for one minute on two topics. First, they were asked to talk about their daily routine on an ordinary weekday. Next, we asked them how they experienced the COVID19 crisis. By surveying two situations, the reliability of the variable could be established. Dependent variable was the number of words spoken per minute (wpm).

#### Articulation speed

We again had two tasks. In the first task, we asked the participants to count from 1 to 10 three times in a row, as fast as possible. This is a well-known sequence of words that should reduce or eliminate speech errors and stop words like ‘er’. The numbers were visible as a single row of Arabic numerals on the computer screen. Participants were told they could use them as a mnemonic device if needed, so that they could fully concentrate on articulating as rapidly as possible. In the second task, we asked participants to say the months of the year twice, also as quickly as possible and consulting a vertical list of months on the computer screen if needed. By having two measurements and asking participants to name the stimuli more than once, we tried to get a more accurate picture of that person’s articulation speed. Dependent variable was the number of words said per minute (wpm).

#### Vocabulary knowledge

Vander Beken et al. (2018) published a vocabulary test for adult Dutch speakers, consisting of 70 words with four response alternatives to choose from. Participants had to indicate which alternative most closely matched the target word. Subsequent Item Response Theory analysis of the original data and a new study indicated that the test could be shortened to 40 items without loss of information (see http://crr.ugent.be/archives/2197). We used the shortened version, which is available in Appendix 2. Dependent variable was the number of correct alternatives selected (on a total of 40).

#### Author recognition test

Participants also completed the Dutch Author Recognition Test (DART_R; Brysbaert et al., 2020). Participants were given a list of 132 names, of which 90 were fiction authors and 42 unknown names. Participants were asked to indicate which authors they knew. They were told that not all names were from book authors and that their score would be reduced if they said they knew one of these non-existing authors. Scores were calculated as follows: (number of authors selected)/ 90 – (number of foils selected)/42. A participant selecting 48 of the 90 authors and one of the foils as an author they know, we would get a score of 48/90 – 1/42 = .51 or 51%. The test is available in Appendix 3.

#### Number of books read in the past year

At the end of the DART, we also asked participants to write down the number of books they had read in the past year.

### Procedure

Participants were sent a link to the online survey, in which we asked the background questions and sought informed consent to participate in the study. Participants were also asked to complete the Dutch author recognition test (DART_R) and the Dutch vocabulary test. There were no time limits for these tests.

Participants who indicated a willingness to take part in the second session, could sign up for an online Zoom session at a particular time through Doodle, the link of which was present in the online survey. Due to the COVID19 measures at the time, it was not possible to invite the participants for testing in person.

At the beginning of the Zoom interview, we checked how the first part of the trial had gone. Permission to record the conversation was sought. We then started measuring reading rate using the 12 texts described above. The texts were presented via Zoom by sharing the computer screen with the participant. They were asked to read the text as they normally would and to indicate when they had finished. The time was registered with a stopwatch. Participants read the first 6 texts silently and the following 6 aloud. Because the conversation was recorded, we could check the time registrations afterwards if there was uncertainty.

Next talking speed was measured. Participants were asked to talk for 1 minute about their daily routine on a normal weekday and 1 minute about how they experienced the COVID19 pandemic. This was also recorded and could be analysed afterwards.

Finally, we checked articulation rate. First, participants were asked to count from 1 to 10 three times, as fast as possible. The numbers were visible on the computer screen. Next, we asked them to say the months of the year twice. Again, these could be read from the computer screen, which was shared via Zoom. Like the measurement for reading speed, we could review this part afterwards and check the time spans in case of uncertainty.

The order of the tasks was fixed so as not to introduce noise that could reduce correlations between tasks. For the same reason, all tasks consisted of the same stimuli for each participant.

## Results

Data and analysis code are available at https://osf.io/t6hsq/?view_only=08ca243944e3475898ddca8d5ff16879.

### Descriptive statistics

Table 1 shows the descriptive statistics.

Reading times were in line with the values reported in Brysbaert (2019) and Brysbaert et al. (2020): 252 words per minute for silent reading (422 syllables per minute),^1^ and 160 wpm for reading aloud (283 syllables per minute). Speech rates when participants were talking about daily routines in their life or about their experiences with COVID19 were slightly higher than reading aloud speed: 176 and 180 wpm, largely because participants used many short words in these descriptions. When talking speed was measured in syllables per minute (spm), speed was slower for talking (247 spm) than reading aloud (283 spm).

Articulation speed was considerably faster for number naming (327 wpm) than for month naming (164 wpm), because the month names are much longer than the names of the numbers 1–10. If we calculate syllables per minute, the patterns reverses: 393 syllables per minute for counting vs. 411 syllables per minute for saying the months, in line with the fact that the syllables of the months are shorter than the syllables of the number names.

### Reliability of the variables

Correlations between variables can only be interpreted if one has information about the reliability of the variables. A low correlation between two variables can be due to a lack of relationship between the variables but also to unreliable measuring of a variable. Reliability was estimated through internal consistency, corrected for length attenuation. For the reading rate measures, this comprised the correlations between the reading rates of the 6 texts. For speaking rate and articulation speed, these were the correlations between the two measurements. For the vocabulary and DART test, reliability was estimated via the correlation between the participant scores on the odd and the even items, because of the distinction made between authors and non-authors.

Reliabilities were estimated with intraclass correlations and Cronbach’s alpha. For intraclass correlations, a distinction is made between ICC2K and ICC3k (Chen et al., 2018). The former gives the value under the assumption that the items are a random sample from possible items; the latter gives the value under the assumption that one is only interested in the items that were presented. ICC2k gives an indication what reliability one can expect in general; ICC3k gives information similar to Cronbach’s alpha.

As can be seen in Table 2, reliability was high (>.8) for the author recognition test, vocabulary size, and reading aloud rate. Reliability was good (>.7) for talking rate, and medium (>.5) for silent reading rate and articulation speed. The difference between ICC2K and ICC3k was particularly large for articulation speed, because the speed was much larger for numbers than for months. The low ICC2k indicates that one will not do well predicting the speed for new stimuli; ICC3K indicates that despite the differences in overall speed between the two types of stimuli, performance of participants correlated reasonably well on numbers and months.

**Table 2:** Reliability of the variables (N = 94).


VARIABLES	RELIABILITY		

	ICC2K	ICC3K	CRONBACH ALPHA

DART	.96	.97	.97

Vocabulary	.87	.89	.89

Silent reading	.54	.55	.55

Reading aloud	.83	.96	.96

Talking rate	.72	.73	.73

Articulation speed	.16	.58	.58


### Correlations between variables

Table 3 shows the core analysis of the data: The correlations between the different variables measured. In order to deal with skewness and outliers in a principled way,^2^ we used Spearman correlations. As df = 92, correlations are significant at p < .05 two-tailed if ρ > .2 (not corrected for multiple testing). Silent reading rate correlated significantly with reading aloud (ρ = .48), vocabulary size (ρ = .34), and DART score/books read (ρ = .21), but not with articulation speed (ρ = –.03). The order remained the same when the lack of reliability of the different scores was taken into account by dividing the observed correlation by the square root of the product of the reliabilities (see the lower left half of Table 3). Oral reading rate correlated significantly with talking speed (ρ = .55), silent reading rate (ρ = .48), and articulation speed (ρ = .28). Again, the order remained the same if the correlations were corrected for lack of reliability.

**Table 3:** Spearman correlations between the different variables (N = 94, df = 92). On the diagonal: reliability of the tests as estimated with Cronbach’s alpha. Upper right half: the observed correlations. Lower left half: correlations corrected for lack of reliability of the variables. Correlations are significant at p < .05 two-tailed if ρ > .2.


	DART	BOOKS	VOC	SILENT	ALOUD	TALKING	ARTICUL.

Age	.41	.14	.72	.13	–.05	–.20	–.16

DART	***.97***	.47	.60	.21	.18	.07	–.14

Books	–	–	.33	.22	.10	.02	–.08

Voc	.65	–	***.89***	.34	.16	.21	–.07

Silent	.29	–	.49	***.55***	.48	.17	–.03

Aloud	.19	–	.17	.66	***.96***	.55	.28

Talking	.08	–	.26	.27	.66	***.73***	.26

Articul.	–.19	–	–.10	–.05	.38	.40	***.58***


### Multiple regression analysis

To examine which combination of variables best predicts reading rate, we ran multiple regression analyses for silent and oral reading rate. Because the correlations are based on Spearman correlations, we used the R package Rfit (Kloke & McKean, 2020). This analysis indicated that silent reading rate was predicted by reading aloud (t(91) = 5.19; p < .001) and vocabulary size (t(91) = 2.81, p < .01; R^2^ = .34). Reading aloud was best predicted by talking speed (t(89) = 5.14; p < .001), articulation speed (t(89) = 2.82, p < .01), silent reading rate (t(89) = 2.52, p < .05), and vocabulary size (t(89) = 2.04, p < .05; R^2^ = .38).

### Cluster analysis

**Figure 1:**
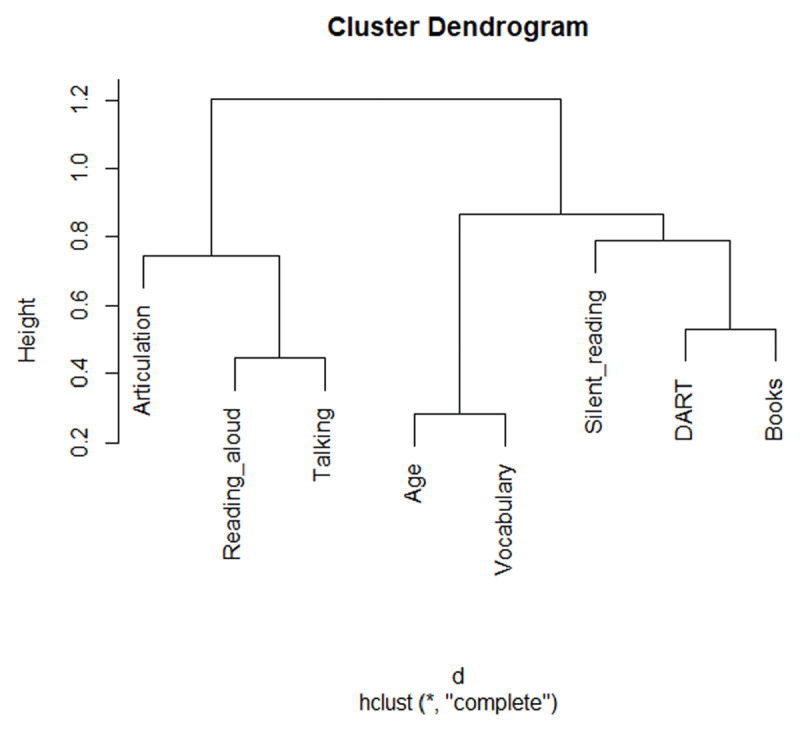
Outcome of cluster analysis on the variables tested, based on Spearman correlations.

Finally, we ran a hierarchical cluster analysis to see how the pattern of correlations is clustered optimally. For this we used the hclust() function of R (version 3.6.2, method = complete). Figure 1 shows the outcome, which splits the variables in two main groups. One group included reading aloud rate together with talking speed and articulation speed. The other cluster comprised silent reading rate with scores of the author recognition test (DART), number of books read in the past year, and vocabulary size. This cluster also included participant age, as older participants knew more words and more authors, and also tended to have a higher silent reading rate. In contrast they had slightly slower talking rates and articulation rates (Table 3).

## Discussion

The present study sought to investigate whether articulation is involved in silent reading. Evidence for this hypothesis was reported by Edfeldt (1959), who measured activity of the articulatory muscles in silent reading, and observed detectable activity in 58 of the 84 participants tested. Counterevidence was provided by Baddeley and Lewis (1981) and Besner (1987), who reported that silent reading is often not hindered by concurrent articulatory movements.

We became interested in the topic after it was discovered that silent reading rate is not twice the speed of speech rate, as was assumed for a long time (Kuperman et al., 2021). As a matter of fact, silent reading rate is not faster than maximum listening rate or maximum articulation speed, making it possible that silent reading involves articulatory codes.

To investigate the possibility, we tested a group of participants on silent reading rate, reading aloud rate, talking rate, and articulation speed. The findings confirmed earlier findings (Brysbaert, 2019). Silent reading rate is well below 300 words per minute (wpm). In the present study it was 252 wpm. Reading aloud is slower (160 wpm) as is talking about familiar topics (175 wpm). However, articulation speed is as fast as silent reading, certainly when differences in syllable length of the stimulus materials are taken into account. Then silent reading rate is 422 syllables per minute (spm), reading aloud rate 283 spm, talking rate 247 spm, and articulation speed 400 spm. So, in principle silent reading is slow enough to allow for activation of the articulatory muscles.

A different picture emerged from the correlational analysis. Whereas there was a statistically significant correlation between oral reading rate and articulation speed (ρ = .28, ρ_corrected_ = .38) and between talking rate and articulation speed (ρ = .26, ρ_corrected_ = .40), there was no significant correlation between silent reading rate and articulation speed (ρ = –.03, ρ_corrected_ = –.05). At the same time, there were significant correlations between silent reading rate and oral reading rate (ρ = .48, ρ_corrected_ = .66) and between silent reading rate and talking rate (ρ = .17, ρ_corrected_ = .27), suggesting that these tasks share common processes (verbal information processing, working memory capacity, cognitive control processes, …). The pattern of correlations indicating that silent reading rate had little in common with articulation speed, was confirmed in multiple regression analysis and a cluster analysis (Figure 1).

Our findings are in line with Besner’s (1987) conclusion that articulation is not involved in silent reading (see also Gregory, 2022, for arguments why inner speech may resemble imagined speech more than real speech).^3^ At the same time, they do not exclude the claim of Baddeley and Lewis (1981) and Edfeldt (1959) that articulation may be used by some participants (in particular less proficient readers) and may become included when participants are asked to read difficult texts. Our findings only indicate that for the participants tested and the texts used (Appendix 1), articulation did not seem to have a discernable role.

One reason for the lack of a correlation between silent reading rate and articulation rate is that articulation and talking become slower when people grow older, whereas silent reading rate stays the same (at least up to the age of 60). Slower speech rate in older participants has been reported before (Tucker et al., 2021), as is the constant silent reading rate in adulthood up to the age of 60 (Aberson & Bouwhuis, 1997; Rodríguez-Aranda, 2003; Warrington et al., 2018). This means that systematically testing participants differing in age may be the ideal way to further investigate under which conditions articulation is absent from silent reading. The present sample was heavily skewed towards young adults.

Our study provides two other noteworthy findings. First, to our knowledge this is the first study that includes all four variables: silent reading rate, oral reading rate, talking rate, and articulation speed. In addition to the findings discussed before, the data show that oral reading rate can be used as an estimate of talking rate (ρ = .55, ρ_corrected_ = .66), which may be interesting for researchers interested in talking speed, as oral reading rate is simpler to measure. Second, the current findings are in line with the reading rate estimates for Dutch (Brysbaert, 2019). In particular, for oral reading this is important information, as there were only three studies available with a total of 161 participants (for silent reading, there were seven studies with a total of 403 participants).

Our study has three limitations. First, we only have information on non-clinical Dutch-speaking adults. It would be interesting to collect data on other languages and populations. Second, we did not measure reading comprehension. We did so for time reasons and because there was face-to-face contact between the experimenter and the participants. This is different from surveys conducted online and anonymously, where questions on reading comprehension are necessary because participants otherwise tend to skip parts of text. A final limitation is the rather low reliability of some of the data. The simplest way to address this limitation is to test more items, but this may require splitting the survey into two one-hour sessions.

## Additional File

The additional file for this article can be found as follows:

10.5334/pb.1189.s1 Appendixes. Appendix 1 to 3.

## Footnotes

Reading rate for the same texts in Brysbaert et al. (2020) was 228 wpm. The most important reason for the higher speed in the present study probably is that the texts were not followed by four yes/no questions as in Brysbaert et al. (2020).

For the number of books read, one participant estimated they read 80 books in the past year. This is an outlier relative to the other participants, but not an outlier in the sense of a measurement error. Such outliers are captured in a uniform way when ranks are used instead of raw values.

A wider question is to what extent the language of thought depends on spoken language: Do we need inner speech to think, reason, and solve problems? Introductions to this literature can be found in Carruthers (2002), De Bleser & Marshall (2005), Baldo et al. (2015), Bourguignon (2022), and Kompa (2023).
