# Supplementary material for: No Correlation Between Articulation Speed and Silent Reading Rate when Adults Read Short Texts
Source: Psychol Belg. 2023 Jul 17;63(1):82–91. doi: 10.5334/pb.1189 (PMC10360968; doi:10.5334/pb.1189)
Supplement: Appendixes. — Appendix 1 to 3. [file pb-63-1-1189-s1.pdf]

Appendix 1: Texts used

Texts 1, 3, 4, 7, 10 and 12 were read silently; texts 2, 5, 6, 8, 9 and 11 were read aloud.

1.

In Nederland en België krijgen kinderen op Kerstdag niet zoveel cadeautjes als wij. Ze gaan op Kerstdag naar de kerk en hebben meer plezier op de dag van Sinterklaas, die op 6 december valt. De avond voordien zoeken ze iets om geschenken in te leggen. Soms is het een mooi gepoetste schoen, soms een bord of een mandje, en soms hangen ze sokken op zoals wij doen. Sinterklaas rijdt op een wit paard of een witte schimmel en de kinderen laten water achter zodat het dier kan drinken en geven iets om te eten. Ze laten een beetje hooi of haver of een wortel of een stuk brood achter. Als ze braaf zijn geweest, vinden ze de volgende morgen dat Sinterklaas snoep, fruit en speelgoed voor hen heeft achtergelaten. Maar als ze stout zijn geweest, vinden ze helemaal niets of misschien zelfs een roede.

2.

De kinderen vertelden over hun kerstvakanties. "We zijn naar Kansas geweest," zei Jack. "Op een dag waren we aan het schaatsen op het meer en toen sneden enkele jongens een gat in het ijs, staken een lucifer aan en er kwam een vuur uit het gat gedurende twee tot drie minuten." "Oh nee," zeiden de anderen, "dat kan toch niet zijn. Water brandt niet." "Maar het is waar dat het water brandde," zei Jack. "Ik zag het." Ze wendden zich tot de lerares om te horen wat zij te zeggen had en zij legde de vreemde gebeurtenis uit. Blijkbaar zijn er aardgasbronnen onder het meer die gas naar boven laten borrelen. Wanneer het meer bevroren is, wordt het gas opgevangen in grote bellen onder het ijs. "Zie je," zei de lerares, "wanneer een gat in het ijs wordt gekapt, zal het ontsnappende gas branden als het aangestoken wordt."

3.

Een grote zwart-gele V2 raket van veertien meter hoog stond in een woestijn in New Mexico. Leeg woog ze vijf ton. Aan boord was acht ton alcohol en vloeibare zuurstof als brandstof. Alles was klaar. Wetenschappers en generaals trokken zich op een afstand terug en zaten gehurkt achter een aardeheuvel. Twee rode fakkels gingen omhoog als signaal om de raket af te vuren. Met geweldig gebrul en de uitbarsting van een vlam steeg de gigantische raket langzaam op en ging toen sneller en sneller. Achter haar hing een vijfentwintig meter lange, gele vlam. Al snel leek de vlam op een gele ster. In een

paar seconden was de raket te hoog om nog gezien te worden, maar de radar volgde haar terwijl ze omhoog ging aan 5.000 km per uur. Een paar minuten nadat de raket afgevuurd was, zag de piloot van een observatievliegtuig dat ze terugkeerde met een snelheid van 3600 kilometer per uur en zich op de aarde stortte, zestig kilometer van het startpunt vandaan.

4.

Op een algemene en vanzelfsprekende manier is de auteur altijd de hoogste autoriteit, omdat het alleen door hem komt dat we de wereld kunnen binnendringen die hij gekozen heeft voor ons te scheppen. Maar dergelijke algemene uitspraken over autoriteit in fictie zijn waardeloos 42 voor zowel de lezer als de schrijver. De legitieme autoriteit van een auteur ligt niet zozeer in het feit dat hij ons vertelt in welke scène we ons bevinden, dat mensen dit of dat gedaan hebben, en dat wat zij deden een bepaalde betekenis had; het ligt eerder in ons ervan te overtuigen dat de scène, de personages, de betekenis, allemaal samen bewegen in een dynamisch patroon waar we in kunnen geloven, los van de persoonlijkheid van de auteur. Het probleem van de auteur is om niet tussen ons en het object of de persoon te staan die hij ons wenst te tonen. Dit probleem blijft bestaan, zelfs als de 'auteur' zichzelf in het verhaal plaatst als verteller in de eerste persoon of als alwetende verteller.

5.

Toen de Spanjaarden Colombia in Zuid-Amerika veroverden, hoorden ze over een Indianenstam die een geweldige rijkdom bezat. Vele jaren eerder had de vrouw van een Indiaans opperhoofd zich in een meer gegooid om een straf te ontlopen en was ze de godin van het meer geworden. Omdat de stam geloofde dat zij de macht had om hen welvarend en zegerijk te maken, maakte de stam een grootse pelgrimstocht naar het meer van Guatavita om de godin te eren en haar cadeaus te brengen telkens als er een nieuw opperhoofd verkozen werd. Als eerste in de stoet kwamen klagende mannen die tekenen van rouw droegen voor de gestorven leider; daarna kwamen mannen getooid met gouden sieraden en smaragden, met veren in hun haar; daarachter liepen de dapperen van de stam in jaguarhuiden; en dan de priesters in zwarte gewaden en hoge hoeden. Uiteindelijk kwamen de edellieden en de hogepriesters, onder wie de nieuwe chef, die op een wagen reed versierd met goud.

6.

Dit is een spel dat Apen Vliegen genoemd wordt en op een regenachtige dag in een klaslokaal kan worden gespeeld. Laat een persoon Baas zijn. Iedereen moet rechtop gaan staan. De Baas staat voor hen, zegt: "Vogels vliegen," en werpt zijn armen omhoog alsof hij vliegt. Dan zegt hij: "Uilen vliegen", en

werpt zijn armen op dezelfde manier omhoog. De andere spelers moeten ook hun armen omhoog gooien. Baas noemt verschillende vogels die vliegen, en zegt dan plotseling: "Apen vliegen," en werpt zijn armen omhoog. Iedereen die meegaat met zijn armen, moet gaan zitten. Baas begint opnieuw en na het noemen van verschillende andere soorten vogels, kan hij plotseling zeggen: "Varkens vliegen." Wie deze keer de armen optilt, moet gaan zitten. Van verschillende dieren kan gezegd worden dat ze vliegen, maar spelers gebruiken apen het meest. De laatste die gaat zitten, kan de Baas zijn voor het volgende spel.

7.

Soms gebeurt grote geschiedenis plotseling en dramatisch. Soms komt ze bijna weerbarstig ons leven binnengeslopen. In veel gevallen zien mensen pas veel later het belang van dergelijke momenten. Dus waren maar weinig mensen opgewonden toen men voor het eerst een atoom splitste. Waarschijnlijk was de eerste kracht die de mens leerde beheersen de mankracht van de mens zelf. Later temde en trainde hij dieren en zorgde hij voor meer kracht, zoals paardenkracht en olifantenkracht. Vervolgens ontwikkelde hij de stoomkracht door 43 hout en kolen te verbranden. Toen leerde hij hoe hij kracht kon halen uit dynamiet en uit het verbranden van kerosine en benzine. Vervolgens leerde de mens een atoom te splijten en de macht ervan te beheersen, en beschikte hij over een kracht die veel groter was dan wat hij kon opwekken door het verbranden van hout, kolen en benzine. Uiteindelijk leverde atoomfusie, die plaatsvindt in de waterstofbom, een nog veel grotere kracht dan kernsplitsing.

8.

Lang voordat drukwerk bestond, zwierven minstrelen van kasteel naar kasteel en zongen daar voor koningen en hun horigen. Hun liederen gingen meestal over het karakter en de dappere daden van een held. Daarbij gebruikten de minstrelen hun verbeelding en voegden mythische daden toe aan het verhaal. Niemand in die tijd probeerde de verhalen op te schrijven, want weinigen konden schrijven. De verhalen werden mondeling overgeleverd, grotendeels op dezelfde manier als de legenden van Amerikaanse Indianen. Maar rond 700 na Christus werden de verhalen met betrekking tot een dappere held, Beowulf, verzameld door een Angelsaksische dichter. Dit gedicht werd sindsdien vertaald in modern Engels, zodat we vandaag kunnen genieten van het eerste epische gedicht in de Engelse literatuur. Beowulf vocht twee vreselijke gevechten uit, één om een koning te redden en één om zijn eigen volk te redden.

9.

Sensorische stoornissen hebben mentale effecten op een persoon die vergelijkbaar zijn met lichamelijke beperkingen. De persoon wordt uitgesloten ten volle te genieten van bepaalde levensfasen. Als één van de zintuigen defect is, kan hij niet waarnemen zoals normale personen dat doen; zijn sensorische inhoud is beperkt of misvormd. Hij is daardoor begrensd in de inhoud van zijn complexe mentale leven. Denken is afhankelijk van zintuiglijke waarneming, je kunt niet nadenken over zintuiglijke waarnemingen die je niet kunt waarnemen. Een volledig kleurenblinde man kan de wereld van kleuren niet kennen zoals andere personen dat doen. Zijn esthetische waardering wordt beperkt door zijn handicap. Een dove persoon kan nog meer lijden. Het ontbreekt hem niet alleen aan de rijke inhoud van geluiden die normale personen waarnemen, maar hij kan ook worden uitgesloten van het ontvangen van informatie die wordt overgebracht door de spraak van andere personen. Het totale effect van deze beperkingen verschilt van persoon tot persoon.

10.

Er wordt gezegd dat de ruimte doordrongen is van ether, een onzichtbare materie waarin energiegolven worden overgedragen. De belangrijkste etherische trillingen voor planten zijn de lichtgolven, omdat alle planten groeien door de werking van licht, dat een stof in hun bladeren omzet in energie. Verschillende planten gedijen echter het best op verschillende variëteiten van wat wij licht noemen. Er is bijvoorbeeld een duidelijk verschil tussen zonlicht en maanlicht, doordat de trillingen van zonlicht in alle richtingen gaan, terwijl de trillingen van maanlicht gepolariseerd zijn en slechts in één richting lopen. Bepaalde planten, zoals de 44 komkommer, gedijen het best in dit gepolariseerde licht. Sinds de ontdekking van dit wetenschappelijke feit werden verschillende experimentele bedrijven opgericht waar licht gepolariseerd wordt om de groei van bepaalde planten te bevorderen.

11.

Een theorie in de wetenschap dient om orde en betekenis aan te brengen in een bepaald gebied van gebeurtenissen. De taak om een reeks gebeurtenissen te ordenen bestaat uit het ontdekken van relaties tussen de gebeurtenissen, of, zoals we zeggen, het vinden van empirische wetten. In de eerste studiefase gebruiken we inductief redeneren om observaties en experimenten te generaliseren. In de natuurwetenschappen is dit proces eenvoudig, want daar kan een wetenschapper eenvoudige observatiesystemen opzetten die het mogelijk maken om een relatief klein aantal variabelen te isoleren en te controleren. Helaas leent het gedrag van levende organismen zich niet zo gemakkelijk tot

vereenvoudiging en controle. De grotere complexiteit van psychologische verschijnselen maakt het experimenteel isoleren en meten van relevante variabelen moeilijk, zoniet onmogelijk. Bijgevolg moeten psychologen veel meer veronderstellen of theoretiseren dan natuurkundigen moesten doen in dit stadium van de ontwikkeling van hun kennisgebied.

12.

De betekenis van de stimuli die moeten geleerd worden is een van de belangrijkste factoren bij verbaal leren. Of verbale betekenis gelijkgesteld kan worden aan verbale context, is een omstreden vraag, maar beide concepten hebben zeker veel gemeen. We kunnen betekenis laten samenvallen met de totaliteit van voorwaarden die het verbale gedrag beïnvloeden, en we kunnen context omschrijven als de zuiver verbale omgeving van de reactie. Binnen het enge kader van verbale leerexperimenten lijkt het daarom redelijk om te spreken van zinloze lettergrepen of cijfers als symbolen met weinig verbale context en veel nieuwe informatie per symbool, terwijl proza of poëzie veel verbale context hebben en weinig nieuwe informatie per woord. Het relatieve gemak waarmee proza of poëzie geleerd kunnen worden weerspiegelt de positieve overdracht van bekende reactiepatronen, terwijl negatieve overdracht een hoogtepunt bereikt wanneer het patroon van symbolen willekeurig of onbekend is.

Appendix 2: Vocabulary test (correct answers in bold)

**Woord**	**Alternative_1**	**Alternative_2**	**Alternative_3**	**Alternative_4**
Romig	Slaperig	Slordig	**Dik en vloeibaar**	Met lijm bedekt
Rekruut	**Soldaat**	Reglement	Onmens	Hoedanigheid
Rekwisieten	Beperkingen	**Benodigdheden**	Afbakeningen	Versnaperingen
Paviljoen	**Bijgebouw**	Bijbedoeling	Bijfiguur	Bijgerecht
Onbekwaam	Aanstootgevend	Niet passend	**Niet geschikt**	Niet bezonnen
Woelig	Tactvol	**Turbulent**	Delicaat	Ontroerd
Schrokop	Domoor	Schroothoop	Vogelschrik	**Gulzigaard**
Kakofonie	Geheimschrift	**Kabaal**	Vuile praat	Signalisatie
Wrok	Bouwval	Keukengerei	**Haat**	Gierigaard
Stramien	Geheim	Moeizaam	**Patroon**	Zeer hoog
Tendens	Aantrekkelijkheid	**Neiging**	Verleiding	Bekoring
Courant	Vloeiend	**Gebruikelijk**	Toegeeflijk	Te voet
Stagnatie	**Stilstand**	Troonsafstand	Wisseling	Aanpassing
Macaber	**Griezelig**	Kleurrijk	Ambitieus	Onbetrouwbaar
Grimeren	Beschadigen	**Beschilderen**	Beschermen	Beschuldigen
Hekelen	Overgieten	Spelen	Inzouten	**Bekritiseren**
Martelaar	Valsaard	Muggenzifter	**Lijder**	Prutser
Prieel	Uit overtuiging	**Tuinhuis**	Oorspronkelijk	Gedeeltelijk
Nerf	Marterachtige	**Bladader**	Zenuwlijder	Sukkel
Perikelen	Rondkijken	Slachten	**Moeilijkheden**	Aanmoedigen
Opsmuk	Opschudding	**Versiering**	Beveiliging	Ontplooiing
Lijvig	Saai	**Dik**	Opwindend	Lichamelijk
Exploitatie	Een niet-democratische staatsvorm	Opgeblazenheid	**Gebruik maken van**	Loslaten van een orgaan
Guitig	Voordelig	Bevorderlijk	Plechtig	**Speels**
Divan	Tuingereedschap	**Meubelstuk**	Auto-onderdeel	Operazangeres
Relaas	**Verslag**	Troost	Steun	Familielid
Onversaagd	Voortreffelijk	**Dapper**	Vrijmoedig	Oprecht
Betichten	Aanvechten	Betreuren	Bedriegen	**Aanklagen**
Slaags	**In gevecht**	Roomsgezind	Zich door niets onderscheidend	Onderdanig
Finesse	Lenigheid	Lichaamsconditie	**Bijzonderheid**	Levendigheid
Wauwelen	Dromen	Schommelen	Spelen	**Babbelen**
Platvloers	Languit	Vlak	**Grof**	Effen
Laakbaar	Niet te vertrouwen	**Afkeurenswaard**	Afschuwwekkend	Aan lijden onderhevig
Patstelling	Positie van waaruit men kan schieten	**Situatie zonder oplossing**	Mening die afwijkt	Uitspraak van een opschepper
Riant	Afwijkend	Grappig	Verzoeningsgezind	**Aantrekkelijk**
Schimpen	Scheuren	**Schelden**	Schudden	Schuiven
Successief	Geslaagd	Zegevierend	Erfelijk	**Achtereenvolgend**
Heling	Aanraken van heilige voorwerpen	**Aannemen van gestolen goed**	Aanmanen tot actie	Aandrijven van voertuigen
Verbolgen	Taboe	Beduusd	Verbluft	**Boos**
Gade	Overtuiging	**Echtgenoot**	Burgerwacht	Klutser

Appendix 3: Dutch Author Recognition Test R (Brysbaert et al., 2020)

Rank	Name	Code
1	E_Buxton	NON
2	Kim_Wassing	NON
3	Marc_De_Bel	AUT
4	Arnon_Grunberg	AUT
5	Simone_van_der_Vlugt	AUT
6	Umberto_Eco	AUT
7	Jorge_Eudoro_Remache	NON
8	Jean_M_Auel	AUT
9	Jane_Austen	AUT
10	Tom_Lanoye	AUT
11	JRR_Tolkien	AUT
12	Willem_Frederik_Hermans	AUT
13	Robert_Teesdale	NON
14	Agatha_Christie	AUT
15	Kathryn_Lightner	NON
16	Haruki_Murakami	AUT
17	Thea_Beckman	AUT
18	JJ_Voskuil	AUT
19	Bart_Moeyaert	AUT
20	Virginia_Woolf	AUT
21	Tomas_Arensman	NON
22	Harry_Mulisch	AUT
23	JD_Salinger	AUT
24	Annie_MG_Schmidt	AUT
25	Karin_Slaughter	AUT
26	Jeff_Kinney	AUT
27	Georges_Roudaut	NON
28	James_Patterson	AUT
29	Gabriel_Garcia_Márquez	AUT
30	Mathijs_L_van_Bueren	NON
31	Esther_Verhoef	AUT
32	Vladimir_Nabokov	AUT
33	Tim_Krabbé	AUT
34	HM_van_der_Grinten	NON
35	Hermann_Hesse	AUT
36	Jane_Jessup	NON
37	Ludwig_Lorenz	NON
38	Herman_Brusselmans	AUT
39	Elizabeth_Wigelsworth	NON
40	Richard_Grigley	NON
41	Lucinda_Riley	AUT
42	Tim_Singler	NON
43	John_Kestley	NON

44	Connie_Palmen	AUT
45	Susan_Smit	AUT
46	Stephenie_Meyer	AUT
47	Roberto_Borsani	NON
48	Mark_Twain	AUT
49	Judith_L_Schecter	NON
50	David_Baldacci	AUT
51	Stieg_Larsson	AUT
52	Carry_Slee	AUT
53	JM_Coetzee	AUT
54	Melanie_Marrero_Morales	NON
55	Santa_Montefiore	AUT
56	Manon_Sikkel	AUT
57	Marcus_Fernandes	NON
58	John_Green	AUT
59	Jostein_Gaarder	AUT
60	Andrée_Oudin	NON
61	Jussi_Adler-Olsen	AUT
62	Elizabeth_George	AUT
63	Marion_Pauw	AUT
64	Eric_Ferey	NON
65	Mahmoud_Abdellah	NON
66	Kettil_Christoffersen	NON
67	AFTh_Van_der_Heijden	AUT
68	Anne_Provoost	AUT
69	Paulo_Coelho	AUT
70	Francine_Oomen	AUT
71	Roy_Leeman	NON
72	Michel_Houellebecq	AUT
73	Paul_van_Loon	AUT
74	Suzanne_Vermeer	AUT
75	Margaret_Atwood	AUT
76	Miguel_de_Cervantes	AUT
77	Konstantin_Ryschkov	NON
78	Elena_Ferrante	AUT
79	Lulu_Wang	AUT
80	Sarah_J_Maas	AUT
81	Khaled_Hosseini	AUT
82	Emily_Oldani	NON
83	Hans_Ulfsson	NON
84	Zofia_Kwiatkowski	NON
85	Griet_Op_de_Beeck	AUT
86	Pieter_Aspe	AUT
87	Theresa_Ziegler	NON

88	E.L._James	AUT
89	Yasushi_Sugawara	NON
90	Patricia_Cornwell	AUT
91	Cees_Nooteboom	AUT
92	Liz_Pichon	AUT
93	George_Eliot	AUT
94	John_Grisham	AUT
95	Carlos_Ruiz_Zafón	AUT
96	Kyra_Appels	NON
97	Bertolt_Brecht	AUT
98	EL_Wilford	NON
99	Emily_Brontë	AUT
100	Tom_Clancy	AUT
101	Helen_Fitzgerald	AUT
102	Kristien_Hemmerechts	AUT
103	Sara_Lakin	NON
104	Emmanuelle_Duvernay	NON
105	Harper_Lee	AUT
106	Chiara_Ricci	NON
107	Saskia_De_Coster	AUT
108	Isabel_Allende	AUT
109	Roald_Dahl	AUT
110	Kelly_Weaver	NON
111	Stephen_King	AUT
112	Dan_Brown	AUT
113	John_Flanagan	AUT
114	Rick_Riordan	AUT
115	Herman_Koch	AUT
116	JK_Rowling	AUT
117	Pablo_Daniel_Gonzalez	NON
118	TC_Boyle	AUT
119	Martijn_van_der_Worp	NON
120	Hendrik_van_Weenen	NON
121	Guy_de_Maupassant	AUT
122	Tonke_Dragt	AUT
123	Astrid_Lindgren	AUT
124	Kader_Abdolah	AUT
125	Suzanne_Collins	AUT
126	John_Punnett	NON
127	Charles_Baudelaire	AUT
128	Dimitri_Verhulst	AUT
129	Pim_Duijster	NON
130	Mark_Robin	NON
131	Annet_de_Jong	AUT

132	Nicci_French	AUT
